# Pruritus Associated With Chronic Kidney Disease: A Comprehensive Literature Review

**DOI:** 10.7759/cureus.5256

**Published:** 2019-07-28

**Authors:** Sanzida S Swarna, Kashif Aziz, Tayyaba Zubair, Nida Qadir, Mehreen Khan

**Affiliations:** 1 Internal Medicine, Sir Salimullah Medical College, Dhaka, BGD; 2 Internal Medicine, Icahn School of Medicine at Mount Sinai, New York, USA; 3 Internal Medicine, Desai Medical Center, Ellicott City, MD, USA; 4 Internal Medicine, Liaquat University of Medical and Health Sciences, Liaquat University Hospital Jamshoro, Hyderabad, PAK; 5 Internal Medicine, George Washington University School of Medicine and Health Sciences, Washington DC, USA

**Keywords:** chronic kidney disease (ckd), uremic pruritus, dialysis, uvb light, tacrolimus

## Abstract

The prevalence of pruritus in chronic kidney disease (CKD) patients has varied over the years, and some studies suggest the prevalence may be coming down with more effective dialysis. Chronic kidney disease-associated pruritus (CKD-aP), previously called uremic pruritus, is a distressing symptom experienced by patients with mainly advanced chronic kidney disease. CKD-aP is associated with poor quality of life, depression, anxiety, sleep disturbance, and increased mortality. The incidence of CKD-aP is decreasing given improvements in dialysis treatments, but approximately 40% of patients with end-stage renal disease experience CKD-aP. While the pathogenesis of CKD-aP is not well understood, the interaction between non-myelinated C fibers and dermal mast cells plays an important role in precipitation and sensory stimulation. Other causes of CKD-aP include metabolic abnormalities such as abnormal serum calcium, parathyroid, and phosphate levels; an imbalance in opiate receptors is also an important factor. CKD-aP usually presents as large symmetric reddened areas of skin, often at night. Managing CKD-aP is a challenge. Research in this area is difficult because most studies are not comparable given their small group samples, study designs, and lack of standardized study measures. The most commonly used treatment is a combination of narrow-band ultraviolet B phototherapy and a μ-opioid receptor antagonist such as naltrexone.

## Introduction and background

Chronic kidney Disease (CKD) is a type of kidney disease in which there is a gradual loss of kidney function over a period of months or years (more than three months). Pruritus is a highly prevalent and common condition in patients with advanced CKD and patients with end-stage renal disease on dialysis. Pruritus is present in about 40% to 84% of patients with end-stage renal disease with variation in its distribution and severity [1]. Chronic kidney disease-associated pruritus (CKD-aP), once called uremic pruritus, mainly affects the face, chest, and limbs, and may be generalized in up to 50% of patients [2]. Pruritus is less common in less advanced stages of kidney disease. CKD-aP can occur without any skin disease or can coexist with xerosis (dry skin) in 50% to 80% patients [3]; or with superimposed complications of excoriation including impetigo, linear crusts, papules, ulcerations, and prurigo nodularis [4]. As Mettang et al. notes, “CKD-aP remains a frequent and compromising symptom in patients with advanced or end-stage renal disease; most therapeutic trials have shown only limited success” [5]. Patients with kidney failure, also called end-stage renal disease, on dialysis, having moderate to severe pruritus, often feel depressed and drained, have a 17% higher mortality rate, and have a poor quality of sleep [6]. According to Mettang and Weisshaar, although there are some hypotheses, the pathophysiology behind pruritus is not completely known. Immune system dysfunction and increasing pro-inflammatory factors are gradually becoming the evident factors in CKD-aP. In the etiology of pruritus, a combination of factors may play an important role [7]. Inflammation and malnutrition are also associated with the genesis of pruritus. Lower serum levels of sodium and higher levels of ferritin were found in pruritic patients compared to nonpruritic patients [8]. For treatment of patients with CKD-aP, gabapentin, systemic μ-receptor antagonists, κ-agonists, and ultraviolet (UV)-phototherapy are currently used [9].

## Review

Methodology

The data for this study were collected by searching for around 100 relevant articles, abstracts, and research papers from web sources such as PubMed and Google Scholar. Two-third of the articles were review articles, and one-third of them were case study reports. The sources mentioned above were used because they are valid and reliable sources to obtain information regarding this topic. A problem faced during data collection was that there were not enough studies from a single database. Following keywords were used for searching the database - "CKD", "kidney", "nephrodermatology", "dermatonephrology", "nephron" and "skin". Therefore, to minimize this problem, multiple databases were used, and the inclusion/exclusion criteria were strictly limited. The criteria used were the following: the subject was human, the article was available in English. CKD is a clinical condition that features impairment of kidney function for more than three months. CKD is associated with many clinical findings, and skin findings are one of the most significant in patients with CKD. Pruritus is the most common skin manifestation among patients with CKD.

Epidemiology

The prevalence of pruritus in CKD patients has varied over the years, and some studies suggest the prevalence may be coming down with more effective dialysis. Whereas the reported prevalence between 1980 and 1993 was from 50% to 90%, subsequent surveys note a lower rate (from 22% to 57%). In a report from one of the major trials, the Dialysis Outcomes and Practice Patterns Study (DOPPS), the prevalence of moderate pruritus persisted constantly at 18% between the years 1996 to 2001 and 2012 to 2015. This study demonstrates the lack of reporting of pruritus by patients and lack of awareness by medical staff. Of patients reporting being severely disturbed by pruritus from 2012 to 2015, 18% used no treatment for pruritus, and 17% did not even report itching to health care staff. Sixty-nine percent of medical directors miscalculated the prevalence of pruritus in their units [10]. 

Clinical features of pruritus in CKD

The clinical presentation of pruritus in patients with CKD varies from person to person. The intensity and spatial distribution also vary among the patients over time. The intensity varies from sporadic discomfort to complete restlessness throughout the day and night. The skin of patients with CKD on hemodialysis with chronic itch looks almost the same as patients without itch [5]. There could be secondary skin changes due to scratching, such as excoriation. Excoriation with or without impetigo can occur as a secondary phenomenon; rarely, prurigo nodularis can also occur. Roughly from 25% to 50% of patients with CKD-aP report concerns about generalized pruritus [11]. In the remaining patients, CKD-aP usually affects the back, face, and forearms, respectively [12]. There are some precipitating and aggravating factors of pruritus: heat, dialysis, stress, cold, physical activity, and showering [13-15]. Usually, patients affected with CKD-aP will have these symptoms for months to years. Several studies have found that duration of dialysis is associated with severity of pruritus.

In the DOPPS, dialysis length of three months or less was associated with moderate to severe pruritus, and those on dialysis for 10 years were less likely to have pruritus in adjusted analyses [16]. The presentation of pruritus in patients with CKD is variable which makes it difficult to differentiate from other causes of pruritus. It has been proven in different studies that CKD-aP is more prevalent in males. In a study of the DOPPS database, male gender was associated with 1.1 greater adjusted odds of having moderate to severe pruritus [6].

Pathophysiology of pruritus in CKD

The multifactorial pathophysiology of pruritus in patients with CKD is not well understood. There are several hypotheses regarding the pathogenesis of CKD-aP, including causes that are immune-mediated, caused by histamine, opioid imbalances, uremic toxins, or hyperparathyroidism, etc. It has been found that immune system dysfunction and elevated proinflammatory cytokines are involved in the pathogenesis of pruritus in patients with CKD [17]. There are other factors also suspected to be related to CKD-aP such as electrolyte imbalance and altered endogenous opioid levels. Though the mechanism is unknown, the perception and perhaps perpetuation of itch seem to have a prominent central nervous system component. Calcium phosphate deposition is also suspected to be associated with generalized pruritus. However, bilateral symmetry with no dermatomal distribution of pruritus is a characterization of CKD-aP [1, 18, 19]. Mettang et al. hypothesized that histamine accumulates in patients with renal failure and classically mediates pruritus and reports that “the concept of histamine causing pruritus is challenged by the absence of typical skin findings like wheals and treatment failure with antihistamine” [17].

Treatment of CKD-aP

While many proposed treatment plans for CKD-aP exist, established treatment options are limited. Therapeutic decision making is confounded because findings from many reports were further invalided by additional studies. Treatment options for uremic pruritus include many topical and systemic therapies such as topical capsaicin cream, emollients, tacrolimus cream, ergocalciferol, gamma-linolenic acid (GLA), gabapentin, μ-opioid receptor antagonist, κ-agonist, and ultraviolet B (UVB). Of these therapies, GLA and UVB have shown the most effectiveness. GLA is an essential fatty acid derived from plant seed oil and has anti-inflammatory properties [20]. Chen et al. tested 17 CKD-aP patients in a six-month randomized placebo-controlled double-blind crossover study with a two-week washout period between treatment phases. They used the visual analog scale (VAS) score ranging from zero to 100 and found the intensity of pruritus decreased from 75 to 30 after using GLA [21]. UVB therapy is another good option for treating uremic pruritus, and it works by inhibiting the T helper-1 and T helper-2 cell-mediated immune response. In a study to assess the effectiveness of UVB therapy on uremic pruritus, 18 patients on hemodialysis with severe pruritus were exposed to UVB or the placebo therapy randomly. Nine of ten participants in the UVB group showed improvement in pruritus compared to two of eight the placebo group. Of those responding to UVB, improvement usually occurred after two to three weeks of treatment [22].

Antihistamines are another good modality of treatment for treating CKD-aP. There are two groups of antihistamine - one group antagonizes histamine receptors, and another group inhibits histamine release from mast cells. Different studies have shown that antipruritic properties of histamine receptors antagonists such as diphenhydramine and hydroxyzine were not successful [20, 23-25]. Several recent studies have shown that uremic pruritus can be successfully treated with mast cell stabilizers. A small study was conducted with five patients with uremic pruritus. Eight weeks of treatment with ketotifen decreased their itching severity significantly [17, 26]. A randomized placebo-controlled double-blind crossover study was conducted by Peer et al. with 15 CKD patients with resistant pruritus (both sets of patients were undergoing hemodialysis) treated with 50 gm naltrexone by mouth. The placebo group was observed for seven days, and a washout period of seven days showed pruritis remarkably improved with naltrexone treatment [27]. Topical tacrolimus is also helpful in treating CKD-aP. In a preliminary study, three patients on peritoneal dialysis with severe pruritus were given 0.03% tacrolimus ointment twice daily for seven days. The patients scored the intensity of their pruritus on the VAS. Two of the three patients reported almost complete resolution; the remaining patient experienced a four-point drop in the VAS score (from seven to three) in relation to the intensity of the itch. κ-opioid receptor agonist can also improve itch symptoms in CKD-aP patients; they work by antagonizing μ-opioid receptors and resolve the morphine-induced itch [28]. Both topical and systemic therapies for pruritus are summarised in Table 1 and Table 2 respectively [29].

**Table 1:** Topical therapies for pruritus CKD - chronic kidney disease

Medication	Dose	Notes
Barrier repair creams/moisturizers/emollients	Not applicable	Low pH products may be particularly useful
Topical corticosteroids	Variable	Not directly antipruritic; may be useful in pruritus due to inflammatory skin dermatoses
Topical calcineurin inhibitors	Tacrolimus 0.03% and 0.1% ointment	Particularly useful in anogenital pruritus, may experience transient burning and stinging
	Pimecrolimus 1% cream	
Doxepin	5% cream	Avoid in children, 20% to 25% risk of sedation
Menthol	1% to 3% cream or lotion	Useful in patients who report cooling as an alleviating factor
Capsaicin	0.025% to 0.1% cream	Particularly useful in neuropathic itch; may experience initial transient burning
Salicylic acid	2% to 6%	Useful in lichen simplex chronicus, avoid in acute inflammatory dermatoses and children
Local anesthetics	Pramoxine 1% to 2.5%	Useful for pruritus on the face and that associated with CKD
	Lidocaine patch 5%	Useful in neuropathic pruritus
	Eutectic mixture of lidocaine 2.5% and prilocaine 2.5%	Useful in neuropathic pruritus
	5% urea + 3% polidocanol	Both moisturizing and anesthetic properties, not available in the United States
	Ketamine 5% or 10% + amitriptyline 5% + lidocaine 5%	Compounded agent; may be useful for various forms of chronic pruritus

**Table 2:** Systemic therapies for pruritus CKD - chronic kidney disease; SNRI - selective neuroepinephrine reuptake inhibitor; SSRI - selective serotonin reuptake inhibitor. * Immunosuppressive agents such as cyclosporine, azathioprine, and mycophenolate mofetil may be beneficial for pruritus in severe atopic dermatitis. ¶ All doses are for adult patients, oral administration, unless noted otherwise.

Medication*	Dose range^¶^	Notes
Antihistamines	Variable depending on the medication	No direct effect on pruritus except in urticaria; sedating antihistamines may be useful through their soporific effects
Antidepressants		
SNRIs	Mirtazapine 7.5 to 15 mg at night	Useful in nocturnal pruritus, may cause increased weight and appetite
SSRIs	Paroxetine 10 to 40 mg per day	Consider in psychiatric patients with pruritus and paraneoplastic pruritus
	Fluvoxamine 25 to 150 mg per day	Consider in psychiatric patients with pruritus and paraneoplastic pruritus
	Sertraline 75 to 100 mg per day	Useful in cholestatic pruritus
µ-opioid receptor antagonists	Naltrexone 25 to 50 mg per day	Useful in patients with cholestatic and CKD-associated pruritus; may cause nausea, vomiting and drowsiness; reverses analgesia and may precipitate acute withdrawal in patients receiving opioid analgesics
κ-opioid receptor agonist/µ-opioid receptor antagonist	Butorphanol 1 to 4 mg intranasally per day	Useful in nocturnal and intractable pruritus; may cause nausea and vomiting as well as drowsiness; may precipitate acute withdrawal in patients receiving opioid analgesics; some potential for abuse due to concomitant weak µ-opioid receptor agonist activity
Selective κ-opioid receptor agonist	Nalfurafine 2.5 to 5 micrograms per day	Useful in CKD-associated pruritus; may cause insomnia; approved in Japan only
Anticonvulsants	Gabapentin 100 to 3600 mg per day	Applies to both: useful in neuropathic pruritus, may cause drowsiness and weight gain; usually given in two to three divided daily doses; dose alteration for renal insufficiency may be needed
	Pregabalin 150 to 300 mg per day	
Substance P antagonist	Aprepitant 80 mg per day	Beneficial in pruritus associated with the Sézary syndrome; expensive
Thalidomide	100 mg at night	Primarily used for prurigo nodularis

## Conclusions

CKD is associated with many dermatological manifestations, including pruritus. Approximately 40% of patients with CKD face pruritus in stage four or five of end-stage renal disease. Its pathogenesis is not clear though there are a few hypotheses. One major hypothesis is immune system dysfunction and elevated proinflammatory cytokines; another is an endogenous opioid hormone imbalance. CKD-aP is predominantly seen in male patients, and it occurs bilaterally in a non-dermatomal distribution. There are a few proposed treatment options for CKD-aP, though they are not proven effective based on the limitations of the different study designs. Commonly used treatments for CKD-aP are UVB light and immunomodulatory drugs such as tacrolimus. Antihistamines and opioid antagonists are also used. In the interest of finding a relation between pruritus and CKD, this paper reviewed older cases to find evidence. After reviewing the cases, it was evident that CKD is associated with the major skin manifestation of pruritus. This paper is limited in that there are few observational or experimental studies conducted on this topic. Therefore, we recommend more studies should be conducted, especially about dermatological manifestations in patients with CKD.
